# A scoping review of measures to assess health professionals’ competencies related to health literacy

**DOI:** 10.1093/heapro/daaf227

**Published:** 2026-01-21

**Authors:** Olivia Mac, Sarah Marshall, Kathleen McFadden, Julie Ayre, Kirsten J McCaffery, Sk Masum Billah, Danielle M Muscat

**Affiliations:** Sydney Health Literacy Lab, Sydney School of Public Health, Faculty of Medicine and Health, The University of Sydney, Room 128c Edward Ford Building (A27), Sydney, NSW 2006, Australia; Sydney Health Literacy Lab, Sydney School of Public Health, Faculty of Medicine and Health, The University of Sydney, Room 128c Edward Ford Building (A27), Sydney, NSW 2006, Australia; Sydney Health Literacy Lab, Sydney School of Public Health, Faculty of Medicine and Health, The University of Sydney, Room 128c Edward Ford Building (A27), Sydney, NSW 2006, Australia; Sydney Health Literacy Lab, Sydney School of Public Health, Faculty of Medicine and Health, The University of Sydney, Room 128c Edward Ford Building (A27), Sydney, NSW 2006, Australia; Sydney Health Literacy Lab, Sydney School of Public Health, Faculty of Medicine and Health, The University of Sydney, Room 128c Edward Ford Building (A27), Sydney, NSW 2006, Australia; Sydney Health Literacy Lab, Sydney School of Public Health, Faculty of Medicine and Health, The University of Sydney, Room 128c Edward Ford Building (A27), Sydney, NSW 2006, Australia

**Keywords:** health literacy, education and training (see medical education and training), medical education and training

## Abstract

Health professionals play an important role in addressing health literacy and ensuring that health systems and information are easy to understand, access, and navigate. We conducted a scoping review to identify and describe measures and tools that assess health professionals’ health literacy competency related to knowledge, skills, practices, attitudes, self-efficacy, and behavioural intentions. Electronic databases MEDLINE, Embase, CINAHL, PubMed, and Scopus were searched in August 2023 and updated in November 2024. Articles were eligible if they included a measure of health professionals’ knowledge, skills, and competencies related to health literacy. We included quantitative studies conducted in all clinical settings and all study types. Two authors independently screened titles, abstracts, and full text articles. The relevant data were extracted and narratively synthesized. In total, 128 articles were identified. We identified 88 unique measures to assess health professionals’ health literacy competencies, which were applied across a range of health professional contexts. Overall 59% (*n* = 76) of tools were purpose-built and used only once. The most frequently assessed domain of health literacy competency was performance-based knowledge, assessed by 45 unique measures. Thirty-seven unique measures reported some form of validity and/or reliability testing, however, of these measures only 16% (*n* = 6) examined construct validity. This review provides a comprehensive overview of existing measures and highlights the need for rigorous and externally valid tools that are more closely aligned with health literacy competency frameworks.

Contribution to health promotion

- Health professionals have an important role to play in making sure health services and health information are easy to access, understand, and navigate.
- We looked at existing measures to assess health professionals’ health literacy competencies (e.g. skills, knowledge, and confidence).
- Most of the measures identified in our review were purpose-built, only used once and not validated.
- Our review highlights the need to improve evaluation work in this area. This will strengthen efforts to make sure health literacy is included in health professional education and practice.

## Introduction

Low health literacy is prevalent worldwide and is associated with poorer health outcomes, including higher mortality, morbidity, medication errors, and rates of hospitalization and emergency department visits (Berkman *et al*. 2011). As definitions of health literacy evolve, they increasingly recognize health literacy as being a product of both ‘personal’ and ‘organizational’ factors, not the sole responsibility of individuals (Nutbeam and Muscat 2021). From the organizational perspective, the extent to which health workers, services, systems, organizations, and policymakers recognize, accommodate, and enable health literacy has been referred to as ‘health literacy responsiveness’ (WHO 2022).

Health professionals have a critical role to play in addressing health literacy and ensuring that health systems and information are easy to understand, access, and navigate in ways that are responsive to patients’ health literacy needs (Nutbeam and Lloyd 2021). It is essential that health professionals have sufficient knowledge and awareness of health literacy, as well as the capabilities and intentions to successfully use health literacy and communication strategies in practice. This is increasingly recognized globally, with many national and international health literacy policies and frameworks acknowledging the importance of an informed and engaged workforce. For example, national health literacy action plans in New Zealand, Germany and the United States of America offer statements relating to enhancing health literacy awareness and capacity among health staff (U.S. Department of Health and Human Services 2010; Ministry of Health 2015).

Several frameworks have been developed to define the specific competencies that health professionals require to support the health literacy needs of consumers from diverse backgrounds and to guide health professional curriculum development (Coleman *et al.* 2013, Beese *et al.* 2025). These competencies emphasize not only individual communication strategies such as using plain language and teachback but also broader professional capabilities such as cultural sensitivity, advocacy, and interprofessional collaboration. This reflects a broader evolution in the construct of competency within health professional education, from discrete technical skills or knowledge to a more holistic assessment integrating knowledge, skills, attitudes, and professional judgement. Assessing health professionals’ health literacy competencies is particularly urgent given the growing complexity of health systems, diversity of patient populations, and post-pandemic demands.

Health professionals have various opportunities to engage with health literacy and contribute to health-literate organizations. Training and education interventions to enhance the competencies of health professionals have also increased over the last decade, demonstrating largely positive outcomes (Coleman 2011; Saunders *et al*. 2019; Talevski *et al*. 2020; Nutbeam and Lloyd 2021). For example, Saunders *et al*. (2019) found improvements in health professional students’ knowledge and self-rated abilities and confidence levels after health literacy training (Saunders *et al*. 2019), and Talevski *et al*. (2020) found training in specific health literacy communication techniques was effective across a wide range of settings, populations, and outcome measures (Talevski *et al*. 2020). Reviews such as these identified a diverse range of evaluation approaches to assess health professionals’ skills, knowledge, practices, and attitudes, which varied in their complexity, methodological rigour, and reliability, highlighting the need for standardized measures.

A recent 2024 scoping review by Nash *et al.* (2024) identified 44 tools assessing aspects of health literacy responsiveness. While useful in highlighting the breadth of tools available, only the name of the identified tool and whether a validation claim was made (yes or no) was presented; other aspects, including the content of the tools, were not analyzed. The proliferation of single-use tools may stem from factors such as the perceived need for context-specific adaptation, limited dissemination of existing solutions, or the lack of centralized repositories for reusable tools. To the authors’ knowledge, there are no other reviews of tools for assessing health professionals’ health literacy competencies. Given the increasing recognition of the critical role health professionals play in addressing health literacy, there is a clear need to examine existing tools in more depth.

This review aimed to identify and describe (i) measures that assess health professionals’ competencies as they relate to health literacy: knowledge, skills, practices, behavioural intentions, attitudes and self-efficacy and (ii) the contexts where such measures have been applied. Guiding questions for this scoping review included:

- What measures exist to assess health professionals’ health literacy competency?
- What domains of health literacy competency are commonly assessed?
- How and in which contexts have measures been applied (e.g. design, countries, and clinical settings)?
- To what extent have existing measures been tested for validity?

## Methods

### Design

Our interest was in mapping the evidence regarding measures that assess health professionals’ health literacy competencies. We chose a scoping review as the approach aligned with our purpose (Munn *et al.* 2018). The review is reported in accordance with the Preferred Reporting Items for Systematic reviews and Meta-Analyses extension for Scoping Reviews (PRISMA-ScR) checklist (Tricco *et al*. 2018). We developed a protocol *a priori*; however, it was not prospectively registered, as the review was exploratory, and its scope evolved during the initial stages.

### Eligibility criteria

To identify measures that assess health professionals’ health literacy competencies, we applied the following inclusion criteria:

- Articles that included a quantitative measure that assessed health professionals’ competencies related to health literacy
- Assessment among health professionals (practicing or in training) from various disciplines and settings
- Varied research designs, e.g. cross-sectional surveys, intervention studies, programme evaluation

Studies were excluded if they met any of the following criteria:

- Included health literacy in a training programme or curriculum but health literacy competencies were not measured as an outcome.
- Related to a specific type of health literacy that focused on particular contexts, conditions, or populations (e.g. mental health literacy).
- Measured the health literacy of health professionals or students (i.e. related to their personal understanding and use of health information and health services) without measuring health literacy competencies (i.e. related to professional capabilities)
- Measured health professionals’ estimates of their patients’ health literacy
- Measured health professionals’ health communication broadly without reference to health literacy

Articles were limited to the English language due to resource constraints and absence of translation capacity. Publication type was limited to primary studies and dissertations, while reviews and commentaries were excluded. In addition, conference proceedings were excluded as they lack sufficient methodological detail for mapping the measures.

### Search strategy

Database searches of MEDLINE, Embase, CINAHL, Scopus, and PubMed were originally conducted in August 2023 and updated in November 2024. The search strategy included concepts of (i) health professionals (e.g. health personnel, clinician, community health provider), (ii) competencies (e.g. knowledge, confidence, attitudes), and (iii) health literacy. We did not include a concept of measurement tool (e.g. instrument, scale, questionnaire) to avoid excluding studies that used these tools but did not focus on them explicitly. The full search strategy for each database is available in Supplementary File 1.

### Study selection process

Search results were uploaded to Covidence, a review management software, and duplicates were removed. Titles and abstracts were independently screened by 2 authors (O.M., K.M., DMM, S.M.) for full text screening. All full texts were retrieved and independently screened (O.M., DMM, S.M.). Inter-reviewer disagreements were resolved through discussion. When consensus could not be reached, a third reviewer was consulted. Once eligible reports were identified, we cross-checked that the primary publication describing the measure development was also included. If the primary publication was not identified in our search, it was added to the included studies.

### Data extraction and synthesis

A data extraction template in Excel was developed to suit the aims of this review. The extraction form was pilot tested with 20 articles and refined. Data were independently extracted by one author and independently checked by another (O.M., DMM, S.M., K.M., T.K., J.A., and S.M.B.). Discrepancies were resolved through discussion with a third author (D.M. or O.M.). Data extracted included (i) description of the study (e.g. study author, year, study aim, setting, type of health professional(s), country), (ii) description of the health literacy competencies measure or items used (e.g. measure name and description, number of items, scoring system, domains of health professional competencies assessed, whether measures were self-reported and/or observer-reported, context of use [cross sectional survey, evaluation of curriculum or evaluation of health literacy training programme]); (iii) measurement properties (e.g. validity and reliability testing, procedures, results), where types of validation were categories as described in Box 1. The domains of health professional competencies were initially developed by the research team, informed by health literacy expertise and understanding of the current literature (Coleman *et al.* 2013, Trezona *et al.* 2017, Cesar *et al.* 2022a, 2022b, Connell *et al.* 2023). Seven domains were iteratively and deductively refined based on the data: knowledge of health literacy (performance-based and self-reported), self-efficacy to address health literacy, direct observation of ability to address health literacy, self-reported frequency of using health literacy techniques, perceived effectiveness and behavioural intentions to address health literacy. Following data extraction, data were presented using frequencies and qualitative descriptions to best summarize the broad evidence identified.

### Ethical approval

Ethical approval was not required for this scoping review.

Box 1 Description of different types of validity testing and methods^a^ **Face validity**

Measures how well a measure/tool or test appears to measure what it claims to (on the surface). Types of methods include expert review and pilot testing.

**Content validity**

Measures how well the content of a measure or tool covers the full range of constructs it intends to measure. Types of methods include expert panel ratings, blueprinting/mapping against constructs and quantitative methods such as content validity index (CVI) and content validity ratio (CVR).

**Construct validity**

Measures the degree to which a measure/tool measures the theoretical construct it intends to. Subtypes of construct validity include discriminant validity, convergent validity, and factorial validity. Types of methods include factor analysis (confirmatory and exploratory), principal component analysis, item response theory, correlation studies, known-group comparisons, and hypothesis testing.

**Criterion validity**

Measures the extent to which test scores are associated with a relevant outcome or external criterion. Subtypes of criterion validity include concurrent validity and predictive validity. Types of methods include sensitivity/specificity analyses, correlation/regression studies and ROC analysis

^a^Based on Messick’s unified theory of validation (Messick 1995).

## Results

### Search results

**Figure 1:**
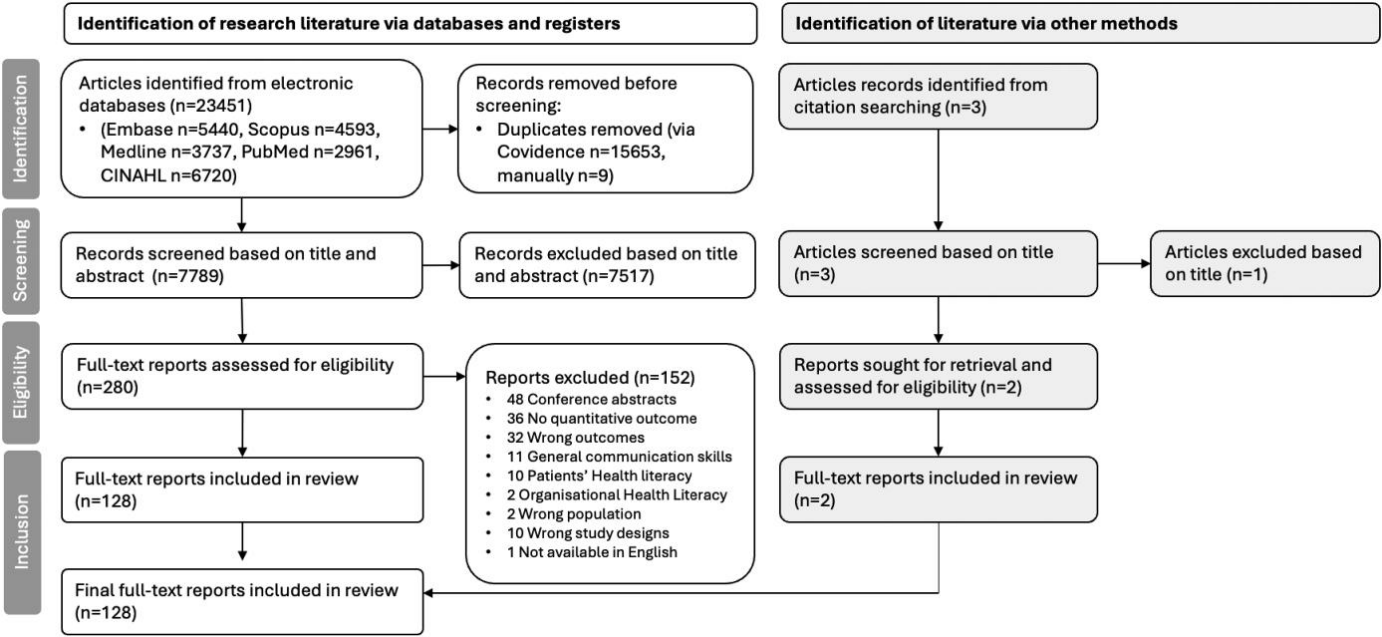
PRISMA flow diagram of search results.

The database searches yielded 7798 unique results. After title and abstract screening, 280 full texts were assessed for eligibility. After full-text screening, 126 articles were eligible for inclusion. Two additional studies were included by citation searching for the primary publication. Figure 1 illustrates the systematic search results. Of the total 128 included studies measuring health professionals’ health literacy competency, we identified 88 unique measures.

### Characteristics of included articles

The characteristics of 128 included studies are summarized in Table 1 and outlined below. By region, most studies were conducted in North America (*n* = 83, 64.8%), with smaller numbers in Asia (*n* = 13, 10.2%), Europe (*n* = 13, 10.2%), Australia (*n* = 9, 7.0%), the Middle East (*n* = 6, 4.7%), and Africa (*n* = 4, 3.1%). By country, the majority of studies were conducted in the United States (*n* = 80, 62.6%). The majority of studies were published after 2016 (*n* = 81, 63.3%), with 40 studies published between 2016 and 2020 and 41 studies published between 2021 and 2024. Almost half of the included studies (*n* = 58, 45.7%) were cross-sectional surveys assessing health professionals’ health literacy competencies. Forty-3 studies (33.9%) conducted evaluations of health literacy training programmes in health organizations and 20 studies (15.7%) evaluated curriculum (i.e. a health literacy training programme embedded into student curriculum). A smaller number of studies (*n* = 5, 3.9%) were focused on the validation and/or translation of existing measures. The bibliographic details of all included studies are displayed in Supplementary Table S1.

**Table 1:** Characteristics of the included studies (*n* = 128).

Study characteristic	Number of studies *n* (%)
**Region**	
North America	83 (65)
Asia	13 (10)
Europe	13 (10)
Australia	9 (7)
Middle East	6 (5)
Africa	4 (3)
**Year of publication**	
≤2010	12 (9)
2011–2015	33 (26)
2016–2020	40 (31)
2021–2024	41 (32)
**Purpose of the study**	
Cross-sectional survey	58 (45)
Evaluation of health literacy training program	43 (34)
Evaluation of curriculum	20 (16)
Validation and/or translation	5 (4)
**Type of health professionals**	
Physicians^a^	37 (29)
Nurses	35 (27)
Allied health professionals^b^	30 (23)
Advanced practice providers^c^	12 (9)
Non-clinical health staff or faculty^d^	11 (9)
Community health care workers	3 (2)
Health professionals (not otherwise specified)	3 (2)
Dentists	2 (2)
Midwives	1 (1)
***Health professional students***	
Nursing students	21 (16)
Allied health professional students^b^	15 (12)
Medical students	8 (6)
Students of health-related degrees (not otherwise specified)	2 (2)
Nurse practitioner students	1 (1)
Dental students	1 (1)

.^a^ Physicians included family medicine residents, GP trainees, GPs, medical residents, medical doctors, internal medicine residents, paediatric residents, physicians, plastic surgeons, orthopaedic surgery trainees, surgeons. ^b^Allied health professionals and students included physiotherapists, pharmacists, psychologists, dental hygienists, osteopaths, occupational therapists, radiation therapists, respiratory therapists, speech pathologists, physical therapists and child life therapists.^c^Advanced practice providers include nurse practitioners and physician assistants. ^d^ Non-clinical health staff included health educators, office staff, medical librarians, administrators, clinic leaders (managers and supervisors) and customer service representatives.

#### RQ1. What measures exist to assess health professionals’ health literacy competencies?

We identified 88 unique measures to assess health professionals’ health literacy competences. Seventy-six measures were purpose-built and used only once. Thirteen measures were used in more than one study (Supplementary Table S2). The Health Literacy Knowledge and Experience Survey (HLKES), developed by Cormier and Kotrlik (2009), was the most frequently used measure, employed in 13 additional studies (14 studies in total). Of these, 7 studies used the original measure with no modifications, 3 studies used a modified version (e.g. addition or removal of items or minor changes to wording) and 3 studies culturally adapted and translated the measure. The HLKES was also separately modified to produce the shortened HLKES-2 (Walker *et al.* 2019) which was culturally adapted and translated in 3 studies. A measure developed by Mackert *et al.* (2011) with no name assigned was used in 8 additional studies (9 studies in total); 3 used the existing measure with no changes, 4 used a modified version, and one study culturally adapted and translated the original measure. The Nursing Professional Health Literacy Questionnaire (Macabasco-O’Connell *et al*. 2011) was used in 3 additional studies. Two studies used a modified version and one study culturally adapted and translated the measure. Two studies used a health literacy questionnaire developed by the Centre of Culture Ethnicity and Health. The Health Literacy Strategies Behavioural Intentions Questionnaire (HLSBIQ) (Cafiero 2013) was used in one additional study. A measure developed by Kaper (2019A) (no name assigned) was used in 2 additional studies; one study used the existing measure with no changes and one study used a modified version. Four measures were only used in one additional study.

#### RQ2. What domains of health literacy competencies are commonly assessed?

The 7 domains of health literacy competencies developed from this review are presented in Table 2. Of the 88 unique measures, performance-based knowledge of health literacy was the most frequently assessed domain, assessed in 45 measures. An example item relating to this domain was ‘Low health literacy levels are prevalent among which of the following age groups…’ (Cormier and Kotrlik 2009). This was followed closely by self-efficacy and self-reported ability to address health literacy, which was assessed by 43 measures. For example, ‘I feel confident in my ability to communicate with patients who have low health literacy*’* (Hildenbrand et al. 2020). Self-reported knowledge was assessed by 21 measures. For example, ‘I understand what it means for patients to have low health literacy’ (Kaper et al. 2019, 2020). Thirty-one measures assessed self-reported frequency of using health literacy and communication techniques. For example, ‘I screen patients for limited health literacy’ (1 = always, 5 = never) (Naperola-Johnson *et al.* 2022). Thirty-four measures assessed perceived effectiveness, attitudes and perceptions of health literacy and/or health literacy techniques. For example, ‘My use of health literacy strategies with patients will result in patients having a better understanding of their illness and its treatment*’* (Cafiero 2013). Behavioural intentions to use health literacy strategies or communication techniques were assessed in 7 measures. For example, ‘I intend to use the teach-back method next time I am with a patient’ (Muscat *et al.* 2021). Lastly, 3 measures included a direct assessment of ability to use health literacy techniques/address health literacy, for example, assessing how well health professionals explained things in plain, non-medical language (Green *et al.* 2014). Twenty-four measures only assessed a single domain, while the majority (*n* = 64) assessed at least 2 domains (Supplementary Table S3). The maximum number of domains assessed by a single measure was 5 (Kelly *et al.* 2020, Shing *et al.* 2023). Self-efficacy and knowledge (both self-reported and performance based) were frequently assessed together.

**Table 2:** Domains of health literacy competencies assessed by unique measures (*n* = 88).

Domain of health literacy competencies	Example items	*n* (%)
Performance-based or objective assessment of health literacy knowledge	Low health literacy is associated with poorer overall health status (Y/N) (Mibei and Daniels 2019)	44 (50)
Self-reported knowledge and awareness of health literacy	I can explain the definition of health literacy (Goto *et al.* 2014, 2015) (7-point Likert, 1 = strongly agree, 7 = strongly disagree)	19 (21)
Self-efficacy (e.g. confidence) and self-reported ability to address health literacy or use health literacy techniques	How confident are you in your ability to effectively communicate with your patients? (Howard *et al.* 2013) (7-point Likert, 1 = not at all confident, 7 = very confident)	43 (49)
Direct observation of ability to use health literacy techniques/address health literacy	Uses appropriate language for patient’s level of understanding and literacy (Wu *et al.* 2020) (Y/N)	3 (3)
Self-reported frequency of using health literacy and communication techniques	How often did you use a health literacy screening tool to assess health literacy? (Cafiero 2013, Anselmann *et al.* 2024) (7-point Likert)	31 (35)
Perceived effectiveness of health literacy techniques and perceptions, attitudes and beliefs of the importance of addressing health literacy.	On a scale from 1 to 5 (1 = not at all important, 5 = extremely important), how important are plain language and clear communication as health literacy strategies? (Milford *et al.* 2016)	36 (41)
Behavioural intentions to use health literacy and communication techniques/address health literacy in future	I intend to use the teach-back method next time I am with a patient (Muscat *et al.* 2021). (Scale from 1–7 with higher scores indicating higher intention.)	6 (7)

#### RQ3. To what extent have existing measures been tested for validity and reliability

Of 88 unique measures identified in this review, 37 (42%) reported some form of validity and/or reliability testing (Supplementary Table S4). The specific validation and reliability testing methods used are reported in Supplementary Table S5.Twenty measures reported content validity testing using qualitative methods such as expert review (*n* = 13) and pilot testing with health staff (*n* = 17) as well as quantitative assessments such Content validity index (CVI) (*n* = 3) and content validity ratio (CVR) (*n* = 1). Six measures reported testing for construct validity using approaches such as factor analysis (*n* = 2), principal component analysis (*n* = 1), item response theory (*n* = 1) and Bartlett's test (*n* = 1). None reported testing criterion validity. Thirty-four (39%) of the 88 measures reported testing for reliability. Of these, 28 measures were tested for internal consistency using Cronbach’s alpha, 2 measures were tested for inter-rater reliability using Cohen’s kappa and one measure was tested for test–retest reliability using intraclass correlation coefficient. Thirteen measures (15%) were tested for reliability in the absence of any validity testing. In addition to the validation of unique measures reported in Supplementary Table S3, several studies reported conducting content and face validity, as well as reliability testing after culturally adapting and translating the HLKES (Chang *et al.* 2020, Parandeh *et al.* 2020) and the HLKES-2 (Al-Fayyadh *et al.* 2022, Angelopoulou *et al.* 2023, Haney and Yoğurtcu 2023).

#### RQ4. In which health professional contexts have existing measures been applied?

The included measures have been applied across a wide range of health professional contexts (Table 1). The health professionals mostly represented were physicians (*n* = 37), nurses (*n* = 35), and allied health professionals (*n* = 30) (Table 1). Other categories included advanced practice providers (nurse practitioners and physician assistants) (*n* = 12), midwives (*n* = 1), dentists (*n* = 2), and community health workers (*n* = 3). The most represented health professional students were nursing students (*n* = 21), allied health professional students (*n* = 15), and medical students (*n* = 8). Eleven studies included non-clinical staff or academic faculty.

## Discussion

This scoping review provides a comprehensive overview of existing measures to assess health professionals’ health literacy competencies. From the 128 studies included in our review, we identified 88 unique measures that were applied across a range of health professional and geographical contexts. This review builds on a recent scoping review by Nash *et al.* (2024), which identified existing tools to assess health literacy responsiveness and explored measurable elements and attributes of health literacy responsiveness. Our review complements this work by describing the domains of health professionals’ health literacy competency assessed by existing tools and by providing a detailed review of the application of existing measures and information on reliability and validity testing.

Our findings show that while a considerable number of measures have been developed, most tools were purpose-built and used only once. Typically, they were tailor made for specific purposes, such as pre-post evaluations of health literacy training programmes or conducting cross-sectional surveys in particular clinical or geographic settings. These tools were not often validated and were often highly context dependent, limiting their generalisability and hindering broader application. However, given that the validation of instruments is resource intensive and requires a specialized skill set, the development and use of these purpose-built measures is not without value. We recognize that there is a need for tools to be adaptable to specific contexts and cultural or geographical differences. To balance the need for standardization and adaptability, future tool development could adopt a tiered or modular design, incorporating core domains while allowing for flexibility and contextual adaptation. This could improve feasibility and use in health professional education. Additionally, most of the studies identified were conducted in North America, primarily in the United States. These factors may limit the generalisability of the findings, particularly to diverse health settings in low- and middle-income countries. Future efforts should prioritize the development and cultural adaptation of tools in low-and middle-income countries. Collaborative approaches and cross-cultural validation studies could further enhance the generalizability and impact of these tools globally.

While knowledge of health literacy (both performance-based and self-reported) and self-efficacy to address health literacy and use health literacy strategies were the most frequently assessed domains, it by no means suggests that these are the most important health literacy competencies. In fact, it may reflect a lack of focus on other key domains of health literacy competencies, such as performance-based assessment of health literacy skills and behavioural intentions to address health literacy. The heavy emphasis on assessing health professionals’ health literacy knowledge may also limit the assessment of more complex competencies such as advocacy, interprofessional collaboration and system-level engagement. These are increasingly recognized as critical components of health literacy competency frameworks, however, are not adequately captured by existing measures. (Coleman *et al.* 2013).

These gaps highlight opportunities to broaden the scope of future assessment tools for health professionals, including the use of alternative methods to assess health literacy competency. For example, simulated encounters, direct observation, video review, and objective structured clinical examinations (OSCEs) may provide more nuanced insights into health professionals’ health literacy competencies. Moreover, the predominance of self-report measures likely reflects their feasibility and ease of administration, however, reliance on self-report introduces the potential for bias, as respondents may inaccurately estimate their knowledge or skills. This, in turn, may limit the ability to assess actual competence or behaviour.

Very few measures underwent rigorous validation testing. Where validation was reported, it was most often limited to face and content validity. While these forms of validation are important, they do not provide sufficient evidence that measures are robust or suitable for application in different contexts (Strauss and Smith 2009), highlighting the need for the development and use of more robust and rigorously validated tools. Importantly, for a scale to be considered a valid instrument, it must demonstrate adequate construct validity as well as reliability (Scholtes *et al.* 2011). In particular, construct and criterion validity are critical to ensure that self-report tools are not only measuring the intended construct (e.g. knowledge or self-efficacy) but are also predictive of actual performance in practice. Future studies should employ established psychometric methods and test tools across various settings, cultural contexts and professional groups to strengthen generalizability. Moreover, there is a need to develop stronger benchmarks for quality health literacy practices. Such benchmarks would allow for meaningful criterion validity. This reflects an important gap, as none of the existing measures tested for criterion validity.

While the majority of measures were used only once, a number of measures were applied in multiple studies and contexts. Of particular note, the HLKES and the related HLKES-2 were frequently applied across a range of health professional and geographical contexts. It is important to note, however, that frequency of use is not necessarily an indicator of quality, and that several frequently used measures were developed before more contemporary conceptualizations of health literacy emerged. For example, the HLKES was published in 2006, prior to recent shifts in emphasis towards organizational health literacy and systems-level change (Sørensen *et al.* 2012, Nutbeam and Muscat 2021). These developments suggest that many existing tools may no longer align well with current understandings of health literacy and its relevance for health professionals. For example, the HLKES focuses primarily on personal health literacy and patient deficits, such as identifying patients at risk of having low health literacy (Cormier and Kotrlik 2009). However, screening for low health literacy is not necessarily an evidence-based practice (Paasche-Orlow and Wolf 2008), with health literacy guidelines recommending adopting a universal precautions approach (Brega *et al.* 2015). Current conceptualisations of health literacy emphasize the critical role of health organizations and healthcare professionals in addressing health literacy and improving the health literacy environment (Koh and Rudd 2015). As such, future measures to assess health professionals’ health literacy competency should be more closely aligned to these conceptualisations.

Alongside the evolution of health literacy conceptualizations, there has also been substantial work to define health literacy competencies and develop competency frameworks for health professional education (Coleman *et al.* 2013, Connell *et al.* 2023, Beese *et al.* 2025).

However, these efforts appear to be disconnected from the development and use of existing measurement tools. Despite the publication of several Delphi consensus studies (Chang *et al.* 2017, Cesar *et al.* 2022a, 2022b, Meyers *et al.* 2024, Beese *et al.* 2025), they do not appear to have informed the content of existing measures. There is a need for more integrated efforts that link health literacy competency frameworks with the development of assessment tools.

Our review highlights the large number of purpose-built and often single use tools that have been developed to assess health professionals’ health literacy competency. A key barrier to the appropriate use of existing tools may be the lack of accessibility of both measures and information about the measures. In addition, the publication type may influence adoption. For example, measures that are published only in journals, especially if behind a paywall, may be less accessible. A centralized repository of measures to assess health professionals’ health literacy competencies, could be a valuable resource for both researchers and educators. Such a platform could house available tools alongside information about their validity and the contexts in which they have been applied. This would be a practical strategy to reduce redundancy, enhance global access and avoid unnecessary development of new, single-use tools. Further, this platform could assist educators and researchers in determining whether existing tools can be applied or adapted to their context before developing single-use tools. This scoping review underscores the importance of establishing comprehensive policies and guidelines to support the systematic development, validation and integration of such tools within accreditation frameworks, such as professional standards, thereby promoting accountability, enabling consistent monitoring of progress, and strengthening the capacity of health systems to deliver health-literate care.

### Strengths and limitations

This is the first review to explore the content, reported validation methods and application of tools for assessing health professionals’ competency related to health literacy. Our novel findings have the potential to advance methodology and consistent reporting of health professionals’ competencies related to health literacy. Our methods were comprehensive and followed best practice guidelines for scoping reviews and our expert-informed, iteratively developed health literacy competencies domains provide a structured framework that others can use to target specific competencies and to adapt or develop new measurement tools to capture these. However, only English-language, peer-reviewed papers were included, which may have introduced publication bias and may limit the global generalizability of our findings. Further, this review may have excluded unpublished organizational tools. Although quality appraisals are not necessary for scoping reviews that aim to map existing evidence rather than evaluate its quality, the absence of these assessments means that the tools identified in this review should be considered individually by researchers planning to use them in practice.

## Conclusion

This review provides a comprehensive overview of existing measures that have been developed to assess health professionals’ health literacy competency and describes the contexts in which these measures have been applied. There is a clear need for international collaboration to create rigorous, theory informed and psychometrically validated tools that are adaptable across diverse professional and cultural contexts. To ensure sustained impact, validated tools should be embedded into accreditation and continuing professional development frameworks. Future efforts should focus on integrating established competency frameworks into the development of standardized tools that are anchored in the core domains of health literacy competencies. Health professionals have a critical role in addressing and improving health literacy. Improving evaluative work in this area will ultimately strengthen efforts to embed health literacy into health professional education and practice.

## Supplementary Material

daaf227_Supplementary_Data

## Data Availability

The data underlying this article will be shared on reasonable request to the corresponding author.
