# Supplementary material for: A scoping review of measures to assess health professionals’ competencies related to health literacy
Source: Health Promot Int. 2026 Jan 21;41(1):daaf227. doi: 10.1093/heapro/daaf227 (PMC12822595; doi:10.1093/heapro/daaf227)
Supplement: daaf227_Supplementary_Data [file daaf227_supplementary_data.zip › SearchStrategy.docx]

| **MEDLINE** | **EMBASE** | **CINAHL** | **Scopus** | **PubMed** |
| --- | --- | --- | --- | --- |
| 1. Knowledge.mp 2. Competenc*.mp 3. Confiden*.mp 4. behavio?r*.mp 5. perception*.mp 6. Awareness.mp 7. Attitude*.mp 8. Health Personnel/ 9. Health Occupations/ 10. Health professional*.mp 11. Health care professional*.mp 12. Medical student*.mp 13. Nurs*.mp 14. Pharmacy*.mp 15. Pharmacist*.mp 16. Doctor*.mp 17. Resident*.mp 18. Dietetic*.mp 19. Clinician*.mp 20. Physician*.mp 21. Pe?diatric*.mp 22. Specialist*.mp 23. Community health provider*.mp 24. Community health worker*.mp 25. Primary care provider*.mp 26. Health provider*.mp 27. Healthcare adminis*.mp 28. Health literacy.tw | 1. Knowledge.mp 2. Competenc*.mp 3. Confiden*.mp 4. behavio?r*.mp 5. perception*.mp 6. Awareness.mp 7. Attitude*.mp 8. health care personnel/ 9. medical profession/ 10. Health professional*.mp 11. Health care professional*.mp 12. Medical student*.mp 13. Nurs*.mp 14. Pharmacy*.mp 15. Pharmacist*.mp 16. Doctor*.mp 17. Resident*.mp 18. Dietetic*.mp 19. Clinician*.mp 20. Physician*.mp 21. Pe?diatric*.mp 22. Specialist*.mp 23. Community health provider*.mp 24. Community health worker*.mp 25. Primary care provider*.mp 26. Health provider*.mp 27. Healthcare adminis*.mp 28. Health literacy.tw | (knowledge OR competenc* OR confiden* OR behavio*r* OR perception* OR awareness OR attitude*)  AND ("health care professional*" OR "health professional*" OR (MH "health personnel+") OR (MH "health occupations+") OR "medical student*" OR nurs* OR pharmacy OR pharmacist* OR resident* OR dietetic* OR pediatric* OR doctor* OR clinician* OR physician* OR specialist* OR "community health provider*" OR "community health worker*" OR "primary care provider*" OR "health provider*" OR "healthcare adminis*")  AND (TI "health literacy" OR AB "health literacy") | (TITLE-ABS-KEY(knowledge) OR TITLE-ABS-KEY(competenc*) OR TITLE-ABS-KEY(confiden*) OR TITLE-ABS-KEY(behavio*r*) OR TITLE-ABS-KEY(perception*) OR TITLE-ABS-KEY(awareness) OR TITLE-ABS-KEY(attitude*))  AND (TITLE-ABS-KEY("health care professional*") OR TITLE-ABS-KEY("health professional*") OR INDEXTERMS("health personnel") OR INDEXTERMS("health occupations") OR TITLE-ABS-KEY("medical student*") OR TITLE-ABS-KEY(nurs*) OR TITLE-ABS-KEY(pharmacy) OR TITLE-ABS-KEY(pharmacist*) OR TITLE-ABS-KEY(resident*) OR TITLE-ABS-KEY(dietetic*) OR TITLE-ABS-KEY(pediatric*) OR TITLE-ABS-KEY(doctor*) OR TITLE-ABS-KEY(specialist*) OR TITLE-ABS-KEY("community health provider*") OR TITLE-ABS-KEY("community health worker*") OR TITLE-ABS-KEY("primary care provider*") OR TITLE-ABS-KEY("health provider*") OR TITLE-ABS-KEY("healthcare adminis*"))  AND TITLE-ABS("health literacy") | (knowledge[tw] or competenc*[tw] or confiden*[tw] or behavio*r*[tw] or perception*[tw] or awareness[tw] or attitude*[tw]) AND ("health care professional*"[tw] or "health professional*"[tw] or "health personnel"[mesh] or "health occupations"[mesh] or "medical student*"[tw] or nurs*[tw] or pharmacy[tw] or pharmacist*[tw] or resident*[tw] or dietetic*[tw] or pediatric*[tw] or doctor*[tw] or physician[tw] or clinician*[tw] or specialist*[tw] or "community health provider*"[tw] or "community health worker"*[tw] or "primary care provider*"[tw] or "health provider*"[tw] or "healthcare adminis*"[tw]) AND "health literacy"[tiab] |

(knowledge[tw] or competenc*[tw] or confiden*[tw] or behavio*r*[tw] or perception*[tw] or awareness[tw] or attitude*[tw]) AND

("health care professional*"[tw] or "health professional*"[tw] or "health personnel"[mesh] or "health occupations"[mesh] or "medical student*"[tw] or nurs*[tw] or pharmacy[tw] or pharmacist*[tw] or resident*[tw] or dietetic*[tw] or pediatric*[tw] or doctor*[tw] or specialist*[tw] or "community health provider*"[tw] or "community health worker"*[tw] or "primary care provider*"[tw] or "health provider*"[tw] or "healthcare adminis*"[tw]) AND "health literacy"[tiab]

(knowledge) or (competenc*) or (confiden*) or (behavio*r*) or (perception*) or (awareness*) or (attitude*)

("health professional*") or ("health care professional*") or INDEXTERMS("health personnel") OR INDEXTERMS("health occupations") or ("medical student*") or (pharmacy*) or (pharmacist*) or (nurs*) or (doctor*) or (clinician*) or (specialist*) or (resident*) or (specialist*) or (physician*) or (dietetic*) or (pe*diatric*) or ("community health provider") or ("community health worker") or ("primary care provider") or ("health provider") or ("health care adminis*")

(TITLE-ABS-KEY(knowledge) OR TITLE-ABS-KEY(competenc*) OR TITLE-ABS-KEY(confiden*) OR TITLE-ABS-KEY(behavio*r*) OR TITLE-ABS-KEY(perception*) OR TITLE-ABS-KEY(awareness) OR TITLE-ABS-KEY(attitude*)) AND
(TITLE-ABS-KEY("health care professional*") OR TITLE-ABS-KEY("health professional*") OR INDEXTERMS("health personnel") OR INDEXTERMS("health occupations") OR TITLE-ABS-KEY("medical student*") OR TITLE-ABS-KEY(nurs*) OR TITLE-ABS-KEY(pharmacy) OR TITLE-ABS-KEY(pharmacist*) OR TITLE-ABS-KEY(resident*) OR TITLE-ABS-KEY(dietetic*) OR TITLE-ABS-KEY(pe*diatric*) OR TITLE-ABS-KEY(doctor*) OR TITLE-ABS-KEY(clinician*) OR TITLE-ABS-KEY(physician*) OR TITLE-ABS-KEY(specialist*) OR TITLE-ABS-KEY("community health provider*") OR TITLE-ABS-KEY("community health worker*") OR TITLE-ABS-KEY("primary care provider*") OR TITLE-ABS-KEY("health provider*") OR TITLE-ABS-KEY("healthcare adminis*")) AND TITLE-ABS("health literacy")
