# Supplementary material for: A scoping review of measures to assess health professionals’ competencies related to health literacy
Source: Health Promot Int. 2026 Jan 21;41(1):daaf227. doi: 10.1093/heapro/daaf227 (PMC12822595; doi:10.1093/heapro/daaf227)
Supplement: daaf227_Supplementary_Data [file daaf227_supplementary_data.zip › Supp_Tables.docx]

**Supplementary Table 1.** Bibliographic details of all included studies

| **First author** | **Year** | **Full reference** |
| --- | --- | --- |
| Al-Fayyadh | 2022 | Al-Fayyadh, S., Al-Jubouri, M. B., Al-Hadrawi, H., Jaafar, S. A., & Hussein, S. M. (2022). Health Literacy-Related Knowledge and Experience among Nurses Practicing in Medical-Surgical Wards. *Nurse Media Journal of Nursing*, *12*(1), 24–31. https://doi.org/10.14710/nmjn.v12i1.42697 |
| Ali | 2014 | Ali, N. K., Ferguson, R. P., Mitha, S., & Hanlon, A. (2014). Do medical trainees feel confident communicating with low health literacy patients?. *Journal of Community Hospital Internal Medicine Perspectives*, *4*(101601396). https://doi.org/10.3402/jchimp.v4.22893 |
| Angelopoulou | 2023 | Angelopoulou, Apostolara, P., Kardari, A., & Velonaki, V.-S. (2023). Nurses’ Knowledge and Experience of Health Literacy: Translation and Validation of the HL-KES 2 Scale in Greek. *International Journal of Caring Sciences*, *16*(3), 1101–1115. |
| Anselmann | 2024 | Anselmann, V., Halder, S., & Sauer, S. (2024). Nursing Students’ Health Literacy and Strategies to Foster Patients’ Health Literacy. *International Journal of Environmental Research and Public Health*, *21*(8). https://doi.org/10.3390/ijerph21081048 |
| Anwar | 2019 | Anwar, M., El-Dahiyat, F., Shriki, H. A., Khaled, M. A., & Rani, A. (2019). An insight into the perception of community pharmacists on health literacy. *Journal of Pharmaceutical Health Services Research*, *10*(4), 433–437. <https://doi.org/10.1111/jphs.12323> |
| Baldocchi | 2013 | Baldocchi, V. (2013). The relationship between registered nurses’ use of health literacy assessment and promotion techniques and their communication self-efficacy*.* (UMI Order AAI3611336) [Northern Illinois University]. |
| Billek-Sawhney | 2012 | Billek-Sawhney, B., Reicherter, E. A., Yatta, B. S., & Duranko, S. G. (2012). Health Literacy: Physical Therapists’ Perspectives. *Internet Journal of Allied Health Sciences & Practice*, *10*(2), 3p–3p. |
| Bilotta | 2012 | Bilotta, D. E. (2012). *Using formative assessment to help nurses learn about limited health literacy.* (UMI Order AAI3613622) [University of Northern Colorado]. |
| Bird | 2022 | Bird, M. L., Elmer, S., Osborne, R. H., Flittner, A., & O'Brien, J. (2022). Training physiotherapists to be responsive to their clients' health literacy needs. *Physiotherapy Theory and Practice*, 38(10), 1398–1406. <https://doi.org/10.1080/09593985.2020.1850956> |
| Boonstra | 2024 | Boonstra M.D., do Amaral M.S.G., Navis G., Stegmann M.E., Westerhuis R., Almansa J., de Winter A.F., & Reijneveld S.A. (2024). Effectiveness of a health literacy intervention targeting both chronic kidney disease patients and health care professionals in primary and secondary care: A quasi-experimental study. *Journal of Nephrology.* <https://doi.org/10.1007/s40620-024-02058-8> |
| Borrero | 2018 | Borrero, J. G. (2018). Nursing Students at the Helm: A Study of the Effect of a Health Literacy Module (HeLM) on the Health Literacy Knowledge, Skills and Attitudes of Pre-licensure Baccalaureate Nursing Students. *CUNY Academic Works* |
| Cafiero | 2012 | Cafiero, M. R. (2012). *Nurse practitioners’ knowledge, experience, and intention to use health literacy strategies in practice.* (UMI Order AAI3508251) [Teachers College, Columbia University]. |
| Cafiero | 2013 | Cafiero, M. (2013). Nurse Practitioners’ Knowledge, Experience, and Intention to Use Health Literacy Strategies in Clinical Practice. *Journal of Health Communication*, *18*(sup1), 70–81. <https://doi.org/10.1080/10810730.2013.825665> |
| Cailor | 2015 | Cailor, S. M., & Chen, A. M. H. (2015). Immediate and longitudinal effects of incorporating health literacy and cultural competency into a yearlong pharmacy curriculum. *Currents in Pharmacy Teaching and Learning*, *7*(3), 292–301. <https://doi.org/10.1016/j.cptl.2014.12.005> |
| Chang | 2017 | Chang, L.-C., Chen, Y.-C., Liao, L.-L., Wu, F. L., Hsieh, P.-L., & Chen, H.-J. (2017). Validation of the instrument of health literacy competencies for Chinese-speaking health professionals. *PLoS One*, *12*(3), e0172859. <https://doi.org/10.1371/journal.pone.0172859> |
| Chang | 2021 | Chang, M. C., Hsieh, J. G., Wei, M. H., Tsai, C. H., Yu, J. H., & Wang, Y. W. (2021). Familiarity, Attitude, and Confidence of Health Literacy Practice among Community Healthcare Providers in Taiwan. *International Journal of Environmental Research and Public Health,* 18(23), 12610. <https://doi.org/10.3390/ijerph182312610> |
| Chang | 2023 | Chang, M. C., Yu, J. H., Hsieh, J. G., Wei, M. H., & Wang, Y. W. (2023). Effectiveness of the refined health literacy course on improving the health literacy competencies of undergraduate nursing students: quantitative and qualitative perspectives. *Medical Education Online*, 28(1), 2173042. <https://doi.org/10.1080/10872981.2023.2173042> |
| Chang | 2020 | Chang, Y. W., Li, T. C., Chen, Y. C., Lee, J. H., Chang, M. C., & Huang, L. C. (2020). Exploring Knowledge and Experience of Health Literacy for Chinese-Speaking Nurses in Taiwan: A Cross-Sectional Study. *International Journal of Environmental Research and Public Health,* 17(20), 7609. <https://doi.org/10.3390/ijerph17207609> |
| Chen | 2020 | Chen A.M.H., Cailor S.M., Wicker E., Harper N.G., Franz T.T., & Pahl B. (2020). Integrating Health Literacy and Cultural Competency Concepts Across the Doctor of Pharmacy Curriculum. *American Journal of Pharmaceutical Education*, *84*(10), ajpe7764. <https://doi.org/10.5688/ajpe7764> |
| Coleman | 2015 | Coleman, C. A., & Fromer, A. (2015). A health literacy training intervention for physicians and other health professionals. *Family Medicine*, *47*(5), 388–392. |
| Coleman | 2016 | Coleman, C.A., Peterson-Perry, S & Bumsted, T. (2016) Long-term effects of a health literacy curriculum for medical students. *Family Medicine, 48(1)* |
| Coleman | 2017 | Coleman, C., Peterson-Perry, S., Sachdeva, B., Kobus, A., & Garvin, R. (2017). Long-term Effects of a Health Literacy Curriculum for Family Medicine Residents. *PRiMER*, *1*. <https://doi.org/10>.22454/PRiMER.2017.703541 |
| Congying | 2024 | Congying, L., Heli, Z., Han, Q., Yang, L., Xiaoling, C., Ruifang, L., & Baohua, L. (2024). Nurses’ health education competence and health literacy: A cross-sectional survey in Chinese county hospitals. *Nurse Education in Practice*, *79*, N.PAG-N.PAG. <https://doi.org/10.1016/j.nepr.2024.104042> |
| Cooper | 2015 | Cooper, L. S., Perryman, M., & Rivers, P. A. (2015). The effects of online health literacy focused training on professional allied health students’ knowledge of health literacy—Part II. *Journal of Health Care Finance*, *41*(4). |
| Cormier | 2009 | Cormier, C. M., & Kotrlik, J. W. (2009). Health literacy knowledge and experiences of senior baccalaureate nursing students. *The Journal of Nursing Education*, *48*(5), 237–248. |
| Cormier | 2016 | Cormier CM. (2006). Health literacy: The knowledge and experiences of senior level baccalaureate nursing students. [Louisiana State University and Agricultural & Mechanical College]. In *Health Literacy: The Knowledge & Experiences of Senior Level Baccalaureate Nursing Students* (UMI Order AAI3244945; p. 137 p). |
| Costitch | 2022 | Costich, M., Bisono, G., Meyers, N., Lane, M., Meyer, D., & Friedman, S. (2022). A Pediatric Resident Curriculum for the Use of Health Literacy Communication Tools. *Health Literacy Research and Practice*, *6*(2), e121–e127. |
| D’Abbondanza, | 2023 | D’Abbondanza, J. A., Roy, M., Okrainec, K., Novak, C. B., von Schroeder, H. P., Urbach, D. R., & McCabe, S. J. (2023). Health literacy awareness among Canadian surgeons. *University of Toronto Medical Journal*, *100*(1), 14–20. ccm. https://doi.org/10.33137/utmj.v100i1.39410 |
| DeBello | 2016 | DeBello, M. C. (2016). The Development and Psychometric Testing of the Health Literacy Knowledge, Application, and Confidence Scale (HLKACS). |
| Devraj | 2010 | Devraj, R., Butler, L. M., Gupchup, G. V., & Poirier, T. I. (2010). Active-learning strategies to develop health literacy knowledge and skills. *American Journal of Pharmaceutical Education*, *74*(8), 137. |
| Devraj | 2011 | Devraj, R., & Gupchup, G. V. (2011). Identifying aspects of pharmacists’ attitudes and barriers toward health literacy: A factor analytic study. *The Annals of Pharmacotherapy*, *45*(6), 771–779. <https://doi.org/10.1345/aph.1P686> |
| Devraj | 2012 | Devraj, R & Gupchup, G. V. (2012) Knowledge of and barriers to health literacy in Illinois. *Journal of the American Pharmacists Association,* 2012;52(6) <https://doi.org/10.1331/JAPhA.2012.12011> |
| Efthymiou | 2023 | Efthymiou, A., Kalaitzaki, A., & Rovithis, M. (2023). Cultural Adaptation of a Health Literacy Toolkit for Healthcare Professionals Working in the Primary Care Setting with Older Adults. Healthcare (Basel, Switzerland), 11(5), 776. <https://doi.org/10.3390/healthcare11050776> |
| Evans | 2014 | Evans, K. H., Bereknyei, S., Yeo, G., Hikoyeda, N., Tzuang, M., & Braddock, C. H. (2014). The Impact of a Faculty Development Program in Health Literacy and Ethnogeriatrics: *Academic Medicine*, *89*(12), 1640–1644. <https://doi.org/10.1097/ACM.0000000000000411> |
| Faruqi | 2015 | Faruqi, N., Lloyd, J., Ahmad, R., Yeong, L.-L., & Harris, M. (2015). Feasibility of an intervention to enhance preventive care for people with low health literacy in primary health care. *Australian Journal of Primary Health*, *21*(3), 321–326. <https://doi.org/10.1071/PY14061> |
| Feinberg | 2021 | Feinberg, I., Hendry, T., Tighe, E., Ogrodnick, M., & Czarnonycz, C. (2021). Teach-Back Training for Nursing Students: Knowledge and Beliefs After Training for Health Literate Communication. *Nursing Education Perspectives*, *42*(3), 177–178. https://doi.org/10.1097/01.NEP.0000000000000670 |
| Fetene | 2024 | Fetene, B. M., Wondiye, H., & Yigzaw, Z. A. (2024). Predictors of Intention to use Health Literacy Strategies in Patient Education among Health Care Professionals at Public Hospitals of Bahir Dar City: Application of Theory of Planned Behavior. *American journal of health promotion,* <https://doi.org/10.1177/08901171241282586> |
| Finlay | 2019 | Finlay, S., Meggetto, E., Robinson, A., & Davis, C. (2019). Health literacy education for rural health professionals: Shifting perspectives. *Australian Health Review : A Publication of the Australian Hospital Association*, *43*(4), 404–407. <https://doi.org/10.1071/AH18019> |
| Galati | 2018 | Galati C., Adams R., Graham K., Reynolds K., & Zametin J. (2018). Health literacy and written communication in skilled nursing/subacute facilities. *OTJR Occupation, Participation and Health*, *38*(2), 131–138. <https://doi.org/10.1177/1539449217723896> |
| Gibson | 2022 | Gibson C., Smith D., & Morrison A.K. (2022). Improving Health Literacy Knowledge, Behaviors, and Confidence with Interactive Training. *Health Literacy Research and Practice*, *6*(2), e113–e120. <https://doi.org/10.3928/24748307-20220420-01> |
| Glick | 2023 | Glick A.F., Farkas J.S., Gadhavi J., Mendelsohn A.L., Schulick N., & Yin H.S. (2023). Pediatric Resident Communication of Hospital Discharge Instructions. *Health Literacy Research and Practice*, *7*(4), e178–e186. <https://doi.org/10.3928/24748307-20230918-01> |
| Goto | 2015 | Goto A., Lai A.Y., & Rudd R.E. (2015). Health literacy training for public health nurses in fukushima: A multi-site program evaluation. *Japan Medical Association Journal*, *58*(3), 69–77. |
| Goto | 2014 | Goto A., Rudd R.E., Lai A.Y., & Yoshida-Komiya H. (2014). Health literacy training for public health nurses in Fukushima: A case-study of program adaptation, implementation and evaluation. *Japan Medical Association Journal*, *57*(3), 146–153. |
| Grabeel | 2018 | Grabeel, K. L., & Beeler, C. J. (2018). Taking the Pulse of the University of Tennessee Medical Center’s Health Literacy Knowledge. *Medical Reference Services Quarterly*, *37*(1), 89–96. <https://doi.org/10.1080/02763869.2017.1404399> |
| Green | 2014 | Green, J. A., Gonzaga, A. M., Cohen, E. D., & Spagnoletti, C. L. (2014). Addressing health literacy through clear health communication: A training program for internal medicine residents. *Patient Education and Counseling*, *95*(1), 76–82. <https://doi.org/10.1016/j.pec.2014.01.004> |
| Griffeth | 2022 | Griffeth E., Sharif I., Caldwell A., Townsend Cooper M., Tyrrell H., & Dunlap M. (2022). Health Literacy Perceptions and Knowledge in Pediatric Continuity Practices. *Health Literacy Research and Practice*, *6*(1), e51–e60. https://doi.org/10.3928/24748307-20220208-01 |
| Groene | 2017 | Groene, O. R., Wills, J., Crichton, N., Rowlands, G., & Rudd, R. R. (2017). The health literacy dyad: The contribution of future GPs in England. *Education for Primary Care : An Official Publication of the Association of Course Organisers, National Association of GP Tutors, World Organisation of Family Doctors*, *28*(5), 274–281. <https://doi.org/10.1080/14739879.2017.1327332> |
| Guner | 2019 | Guner, M. D., & Ekmekci, P. E. (2019). A Survey Study Evaluating and Comparing the Health Literacy Knowledge and Communication Skills Used by Nurses and Physicians. *Inquiry : A Journal of Medical Care Organization, Provision and Financing*, *56*(0171671) <https://doi.org/10.1177/0046958019865831> |
| Gupta | 2020 | Gupta A., Wood M., Kumar S., Misra S., & Turner T. (2020). No Faculty Required: Use of a Health Literacy Low Inference Self-Assessment Measure to Promote Behavior Change. *Academic Pediatrics*, *20*(5), 712–720. <https://doi.org/10.1016/j.acap.2020.02.019> |
| Ha | 2014 | Ha, H., & Lopez, T. (2014). Developing health literacy knowledge and skills through case-based learning. *American Journal of Pharmaceutical Education*, *78*(1). <https://doi.org/10.5688/ajpe78117> |
| Haney | 2023 | Haney, M. Ö., & Yoğurtcu, H. (2023). Psychometric properties of the Health Literacy Knowledge and Experience Survey-2 for Turkish nursing students. *Health Promotion International*, *38*(5), 1–10. <https://doi.org/10.1093/heapro/daad117> |
| Harrington | 2016 | Harrington, M., & Engelke, M. K. (2016). Health Literacy: Perceptions and Experiences of Pediatric Nephrology Interprofessional Team Members. *Nephrology Nursing Journal : Journal of the American Nephrology Nurses’ Association*, *43*(1), 15–26. |
| Hartman | 2014 | Hartman, E. (2014). *Nurses lack skills to teach: Increasing undergraduate nursing skills related to patient education.* (UMI Order AAI3611783) [Capella University]. ccm. |
| Hildenbrand | 2020 | Hildenbrand, G. M., Perrault, E. K., & Keller, P. E. (2020). Evaluating a Health Literacy Communication Training for Medical Students: Using Plain Language. *Journal of Health Communication*, *25*(8), 624–631. <https://doi.org/10.1080/10810730.2020.1827098> |
| Howard | 2013 | Howard, T., Jacobson, K. L., & Kripalani, S. (2013). Doctor talk: Physicians’ use of clear verbal communication. *Journal of Health Communication*, *18*(8), 991–1001. <https://doi.org/10.1080/10810730.2012.757398> |
| Hsieh | 2022 | Hsieh, J.-G., Yu, J.-H., Wang, Y.-W., Wei, M.-H., Chang, M.-C., Wu, C.-C., & Chia, S.-L. (2022). Health literacy training program for community healthcare providers using hybrid online team-based learning in Taiwan. *BMC Medical Education*, *22*(1), 576. <https://doi.org/10.1186/s12909-022-03646-7> |
| Jukkala | 2009 | Jukkala A, Deupree JP, & Graham S. (2009). Knowledge of limited health literacy at an academic health center. *Journal of Continuing Education in Nursing*, *40*(7), 298–304. https://doi.org/10.3928/00220124-20090623-01 |
| Kaper | 2019a | Kaper, M. S., Reijneveld, S. A., van Es, F. D., de Zeeuw, J., Almansa, J., Koot, J. A. R., & de Winter, A. F. (2019). Effectiveness of a Comprehensive Health Literacy Consultation Skills Training for Undergraduate Medical Students: A Randomized Controlled Trial. *International Journal of Environmental Research and Public Health*, *17*(1). <https://doi.org/10.3390/ijerph17010081> |
| Kaper | 2018 | Kaper, M. S., Sixsmith, J., Koot, J. A. R., Meijering, L. B., Van Twillert, S., Giammarchi, C., Bevilacqua, R., Barry, M. M., Doyle, P., Reijneveld, S. A., & De Winter, A. F. (2018). Developing and pilot testing a comprehensive health literacy communication training for health professionals in three European countries. *Patient Education and Counseling*, *101*(1), 152–158. <https://doi.org/10.1016/j.pec.2017.07.017> |
| Kaper | 2019b | Kaper, M. S., Winter, A. F. de, Bevilacqua, R., Giammarchi, C., McCusker, A., Sixsmith, J., Koot, J. A. R., & Reijneveld, S. A. (2019). Positive Outcomes of a Comprehensive Health Literacy Communication Training for Health Professionals in Three European Countries: A Multi-centre Pre-post Intervention Study. *International Journal of Environmental Research and Public Health*, *16*(20). <https://doi.org/10.3390/ijerph16203923> |
| Kelly | 2020 | Kelly, T., Arnold, B., Surjan, Y., Rinks, M., & Warren-Forward, H. (2020). Radiation therapist health literacy training: A qualitative study exploring perceived barriers and attitudes. *Radiography*, *26*(4), 294–301. <https://doi.org/10.1016/j.radi.2020.02.007> |
| Kennard | 2017 | Kennard, D. (2017). Emergency Room Nurses Knowledge of and Experience with Health Literacy and Their Patient Teaching Methods. *Seton Hall University Dissertations and Theses (ETDs). 2270.* |
| Kerr | 2016 | Kerr, M. (2016). Teaching Strategies to Prepare Prelicensure Nursing Students to Perform the Skill of Teach-Back. *Teaching Strategies to Prepare Prelicensure Nursing Students to Perform the Skill of Teach-Back*, *Ph.D.*, |
| Kershner | 2024 | Kershner, S. H., George, T. P., & DeCristofaro, C. (2024). Incorporating Educational Strategies to increase Nurse Practitioner Students’ Understanding of Health Literacy. *Journal for Nurse Practitioners*, *20*(9), <https://doi.org/10.1016/j.nurpra.2024.105177> |
| Kim | 2024 | Kim, K., Metzger, A. M., Win, M. T., Luck, M., & Alrasheed, M. (2024). Knowledge and Perceptions of Health Literacy among Pharmacists in Ohio in 2013 and 2021. *Journal of the American Pharmacists Association : JAPhA*, 102260. <https://doi.org/10.1016/j.japh.2024.102260> |
| Knight | 2011 | Knight, G. D. (2011). *An Evaluation of the Health Literacy Knowledge and Experience of Registered Nurses in Georgia.* (UMI Order AAI3464456) [Auburn University]. |
| Koduah | 2021 | Koduah, A. O., Amoah, P. A., Nkansah, J. O., & Leung, A. Y. M. (2021). A Comparative Analysis of Student and Practising Nurses’ Health Literacy Knowledge in Ghana. *Healthcare (Basel, Switzerland)*, *9*(1). <https://doi.org/10.3390/healthcare9010038> |
| Koenig | 2019 | Koenig, V. E., & Provident, I. M. (2019). Workshop series for occupational therapists using the US Agency for Healthcare Research and Quality’s Health Literacy Universal Precautions Toolkit and other supported tools. *Health Education Journal*, *78*(4), 451–463. <https://doi.org/10.1177/0017896918820067> |
| Logan | 2007 | Logan, R. A. (2007). Clinical, classroom, or personal education: Attitudes about health literacy. *Journal of the Medical Library Association*, *95*(2), 127-e48. |
| Macabasco-O’Connell | 2011 | Macabasco-O’Connell A. & Fry-Bowers E.K. (2011). Knowledge and perceptions of health literacy among nursing professionals. *Journal of Health Communication*, *16 Suppl 3* 295–307. |
| Mackert | 2011 | Mackert, M., Ball, J., & Lopez, N. (2011). Health literacy awareness training for healthcare workers: Improving knowledge and intentions to use clear communication techniques. *Patient Education and Counseling*, *85*(3), e225–e228. <https://doi.org/10.1016/j.pec.2011.02.022> |
| Maduramente | 2019 | Maduramente, T. S., Orendez, J. D., Saculo, J. A., Trinidad, A. L. A., & Oducado, R. M. F. (2019). Health literacy: knowledge and experience among senior students in a nursing college. *Indonesian Nursing Journal of Education & Clinic (INJEC)*, *4*(1). <https://doi.org/10.24990/injec.v4i1.227> |
| Martin | 2023 | Martin, R., Cartwright, J., & Bird, M.-L. (2023). The Health Literacy of First Year Physiotherapy and Speech Pathology Students and Their Perceived Future Roles in Supporting Their Clients’ Health Literacy. *International Journal of Environmental Research and Public Health*, *20*(11). <https://doi.org/10.3390/ijerph20116013> |
| McCleary-Jones, | 2012 | McCleary-Jones, V. (2012). Assessing nursing students’ knowledge of health literacy. *Nurse Educator*, *37*(5). <https://doi.org/10.1097/NNE.0b013e318262ead3> |
| McCune | 2010 | McCune, R. L. (2010). *Assessing Health Literacy in Diverse Primary Care Settings.* (UMI Order AAI3441349) [University of Michigan]. |
| Mibei | 2018 | Mibei, F., & Daniels, F. (2018). Health literacy knowledge and experiences of nursing students at a South African university. *Africa Journal of Nursing and Midwifery*, *20*(2). <https://doi.org/10.25159/2520-5293/3205> |
| Mihalopoulos | 2013 | Mihalopoulos C.C., Powers M.F., Lengel A.J., & Mangan M.N. (2013). Impact of a health literacy training course on community pharmacists’ health literacy knowledge and attitudes. *Journal of Pharmacy Technology*, *29*(6), 283–289. <https://doi.org/10.1177/8755122513502455> |
| Milford | 2016 | Milford E., Morrison K., Teutsch C., Nelson B.B., Herman A., King M., & Beucke N. (2016). Out of the classroom and into the community: Medical students consolidate learning about health literacy through collaboration with Head Start. *BMC Medical Education*, *16* <https://doi.org/10.1186/s12909-016-0635-z> |
| Mnatzaganian | 2017 | Mnatzaganian C., Fricovsky E., Best B.M., & Singh R.F. (2017). An Interactive, Multifaceted Approach to Enhancing Pharmacy Students’ Health Literacy Knowledge and Confidence. *American Journal of Pharmaceutical Education*, *81*(2), 32. <https://doi.org/10.5688/ajpe81232> |
| Mohamed-Yassin | 2023 | Mohamed-Yassin, M.-S., Daher, A. M., Ramli, A. S., Ramli, N. F., & Baharudin, N. (2023). Health literacy-related knowledge, attitude, perceived barriers, and practice among primary care doctors in Malaysia. *Scientific Reports*, *13*(1). <https://doi.org/10.1038/s41598-023-47242-1> |
| Mor-Anvay | 2021 | Mor-Anavy, S., Lev-Ari, S., & Levin-Zamir, D. (2021). Health Literacy, Primary Care Health Care Providers, and Communication. *Health Literacy Research and Practice*, 5(3), e194–e200. <https://doi.org/10.3928/24748307-20210529-01> |
| Munangatire | 2022 | Munangatire, T., Tomas, N., & Mareka, V. (2022). Nursing students' understanding of health literacy and health practices: a cross-sectional study at a university in Namibia. *BMC Nursing*, 21(1), 8. <https://doi.org/10.1186/s12912-021-00776-z> |
| Murphy | 2023 | Murphy, P. Z., & Jester, A. P. (2023). Pharmacists’ Knowledge and Perceptions of Health Literacy. *Journal of Pharmacy Practice*, *36*(3), 620–627. https://doi.org/10.1177/08971900221074958 |
| Muscat | 2021 | Muscat D.M., Ceprnja D., Hobbs K., Gibson J.-A., Blumenthal C., Milad R., Burns C., Lau T., & Flood V. (2021). Development and evaluation of a health literacy training program for allied health professionals: A pre-post study assessing impact and implementation outcomes. *Health Promotion Journal of Australia 32*(Supplement 1), 88–97. <https://doi.org/10.1002/hpja.350> |
| Nantsupawat | 2020 | Nantsupawat A., Wichaikhum O.-A., Abhicharttibutra K., Kunaviktikul W., Nurumal M.S.B., & Poghosyan L. (2020). Nurses’ knowledge of health literacy, communication techniques, and barriers to the implementation of health literacy programs: A cross-sectional study. *Nursing & Health Sciences*, *22*(3), 577–585. <https://doi.org/10.1111/nhs.12698> |
| Naperola-Johnson | 2022 | Naperola-Johnson, J., Gutierrez, J., Doyle, K., Thompson, J., & Hendrix, C. (2022). Implementation of health literacy training for clinicians in a federally qualified health center. *PEC Innovation*, *1*(9918367980406676), 100083. <https://doi.org/10.1016/j.pecinn.2022.100083> |
| Nepps | 2023 | Nepps, P., Lake, A., Fox, J., Martinez, C., Matsen, P., & Zimmerman, K. (2023). Improving Health Equity Through Health Literacy Education. *Health Literacy Research and Practice*, *7*(2), e99–e104. <https://doi.org/10.3928/24748307-20230522-01> |
| Nesari | 2019 | Nesari, M., Olson, J. K., Nasrabadi, A. N., & Norris, C. (2019). Registered Nurses’ Knowledge of and Experience with Health Literacy. *Health Literacy Research and Practice*, *3*(4), e268–e279. <https://doi.org/10.3928/24748307-20191021-01> |
| Newton | 2018 | Newton, L., & Kwekkeboom, K. L. (2018). Improving Postoperative Teaching Through the Use of “Teach-Back”. *ORL-Head & Neck Nursing*, *36*(2), 7–14. |
| Ogrodnick | 2020 | Ogrodnick, M. M., Feinberg, I., Tighe, E., Czarnonycz, C. C., & Zimmerman, R. D. (2020). Health-Literacy Training for First-Year Respiratory Therapy Students: A Mixed-Methods Pilot Study. *Respiratory Care*, *65*(1), 68–74. <https://doi.org/10.4187/respcare.06896> |
| Ogrodnick | 2021 | Ogrodnick, M., O’Connor, M. H., & Feinberg, I. (2021). Health Literacy and Intercultural Competence Training. *Health Literacy Research and Practice*, *5*(4). https://doi.org/10.3928/24748307-20210908-02 |
| O’Neal | 2013 | O’Neal, K. S., Crosby, K. M., Miller, M. J., Murray, K. A., & Condren, M. E. (2013). Assessing health literacy practices in a community pharmacy environment: Experiences using the AHRQ Pharmacy Health Literacy Assessment Tool. *Research in Social & Administrative Pharmacy*, *9*(5), 564–596. <https://doi.org/10.1016/j.sapharm.2012.09.005> |
| Pagels | 2015 | Pagels, P., Kindratt, T., Arnold, D., Brandt, J., Woodfin, G., & Gimpel, N. (2015). Training Family Medicine Residents in Effective Communication Skills While Utilizing Promotoras as Standardized Patients in OSCEs: A Health Literacy Curriculum. *International Journal of Family Medicine*, *2015*, 1–9. <https://doi.org/10.1155/2015/129187> |
| Palesy | 2020 | Palesy, D., & Jakimowicz, S. (2020). Health literacy support for Australian home-based care recipients: A role for homecare workers? *Home Health Care Services Quarterly*, *39*(1). <https://doi.org/10.1080/01621424.2019.1691698> |
| Papa | 2023 | Papa, R., Sixsmith, J., Giammarchi, C., Lippke, S., McKenna, V., Di Furia, L., Ceravolo, M. G., & De Winter, A. (2023). Health literacy education at the time of COVID-19: development and piloting of an educational programme for university health professional students in 4 European countries. *BMC Medical Education*, 23(1), 650. <https://doi.org/10.1186/s12909-023-04608-3> |
| Parandeh | 2020 | Parandeh, A., Rahmati-Najarkolaei, F., & Isfeedvajani, M. S. (2020). Health literacy knowledge and experience survey: Cross-cultural adaptation and the psychometric properties of the Iranian nurse version. *Journal of Education and Health Promotion*, *9*(101593794), 244. <https://doi.org/10.4103/jehp.jehp> |
| Payne | 2009 | Payne, L. (2009). *Registered nurses’ use of teaching techniques to mitigate low health literacy: Frequency, perceived effectiveness and correlations.* (UMI Order AAI3388728) [University of Houston]. |
| Potter | 2007 | Potter, J. (2017). Health Literacy of Nursing Students and Their Awareness of Patient Literacy Needs. *Health Literacy of Nursing Students & Their Awareness of Patient Literacy Needs.* [Walden University] |
| Rajah | 2017 | Rajah, R., Hassali, M. A., & Lim, C. J. (2017). Health Literacy-Related Knowledge, Attitude, and Perceived Barriers: A Cross-sectional Study among Physicians, Pharmacists, and Nurses in Public Hospitals of Penang, Malaysia. *Frontiers in Public Health*, *5*(101616579), 281 <https://doi.org/10.3389/fpubh.2017.00281> |
| Roberts | 2012 | Roberts D.M., Reid J.R., Conner A.L., Barrer S., Miller K.H., & Ziegler C. (2012). A Replicable Model of a Health Literacy Curriculum for a Third-Year Clerkship. *Teaching and Learning in Medicine*, *24*(3), 200–210. <https://doi.org/10.1080/10401334.2012.692261> |
| Ronan | 2023 | Ronan, M., Eagleson, K., Fielden, P., Sutton, N., Gilchrist, S., Schilling, S., Scaini, L., & Ullman, A. (2023). Health literacy in paediatric healthcare: A cross-sectional survey of nurses. *Journal of Children & Young People’s Health*, *4*(2), 5–11. <https://doi.org/10.33235/jcyph.4.2.5-11> |
| Ruggeri | 2021 | Ruggeri B., Vega A., Liveris M., St George T.E., & Hopp J. (2021). A Strategy for Teaching Health Literacy to Physician Assistant Students. *Health Literacy Research and Practice*, *5*(1), e70–e77. <https://doi.org/10.3928/24748307-20210201-01> |
| Sand-Jecklin | 2010 | Sand-Jecklin, K., Murray, B., Summers, B., & Watson, J. (2010). Educating Nursing Students about Health Literacy: From the Classroom to the Patient Bedside. *OJIN: The Online Journal of Issues in Nursing*, *15*(3). <https://doi.org/10.3912/OJIN.Vol15No03PPT02> |
| Schlichting | 2007 | Schlichting, J. A., Quinn, M. T., Heuer, L. J., Schaefer, C. T., Drum, M. L., & Chin, M. H. (2007). Provider perceptions of limited health literacy in community health centers. *Patient Education and Counseling*, *69*(1–3), 114–120. |
| Schwartzberg | 2007 | Schwartzberg, J. G., Cowett, A., VanGeest, J., & Wolf, M. S. (2007). Communication techniques for patients with low health literacy: A survey of physicians, nurses, and pharmacists. *American Journal of Health Behavior*, *31 Suppl 1*(9602338, dz5) |
| Sharifirad | 2015 | Sharifirad, G., Mostafavi, F., Reisi, M., Mahaki, B., Javadzade, H., Heydarabadi, A. B., & Esfahani, M. N. (2015). Predictors of nurses’ intention and behavior in using health literacy strategies in patient education based on the theory of planned behavior. *Materia Socio-Medica*, *27*(1), 22–26. <https://doi.org/10.5455/msm.2014.27.22-26> |
| Shing | 2023 | Shing E.Z., Wally M.K., Seymour R.B., Patt J.C., & Scannell B.P. (2023). Health Literacy Awareness Among Orthopaedic Surgery Residents: A COERG Survey. *JBJS Open Access*, *8*(3), 23.00027. <https://doi.org/10.2106/JBJS.OA.23.00027> |
| Sicat | 2005 | Sicat, B. L., & Hill, L. H. (2005). Enhancing student knowledge about the prevalence and consequences of low health literacy. *American Journal of Pharmaceutical Education*, *69*(4), 460–466. <https://doi.org/10.5688/aj690462> |
| Sriyanah | 2021 | Sriyanah, N., Kadar, K.S & Efendi, S. (2021) Knowledg, attitudes and barriers towards health literacy among health professionals working in hospiteal. *Enfermería Clínica,* 31. <https://doi.org/10.1016/j.enfcli.2021.07.004> |
| Stone | 2021 | Stone, M., Bazaldua, O., & Morrow, J. (2021). Developing Health Literacy Communication Practices for Medical Students. *MedEdPORTAL: The Journal of Teaching and Learning Resources,* 17, 11091. <https://doi.org/10.15766/mep_2374-8265.11091> |
| Stone | 2023 | Stone, M., Bazaldua, O., Piernik-Yoder, B., Sculley, R., & Kosub, K. (2023). A Novel Simulation Program for Interprofessional Health Literacy Training. *Health Literacy Research and Practice*, 7(3), e139–e143. <https://doi.org/10.3928/24748307-20230713-01> |
| Suarez-Balcazar | 2024 | Suarez-Balcazar, Y., Allen-Meares, P., Dickens, C., Brazil, E., Garcia-Bedoya, O., & Biggers, A. (2024). Enhancing the Education of Community Health Workers on Health Literacy and Cultural Humility in Times of COVID-19. *American Journal of Health Education*. <https://doi.org/10.1080/19325037.2024.2366453> |
| Sullivan | 2011 | Sullivan, M. F., Ferguson, W., Haley, H. L., Philbin, M., Kedian, T., Sullivan, K., & Quirk, M. (2011). Expert communication training for providers in community health centers. *Journal of Health Care for the Poor and Underserved*, 22(4), 1358–1368. <https://doi.org/10.1353/hpu.2011.0129> |
| Szwajcer | 2014 | Szwajcer, A., Macdonald, K & Kvern, B. (2014). Health literacy training for family medicine residents. *Journal of the Canadian Health Libraries Association.* 35(3). <https://doi.org/10.5596/c14-033> |
| Torres | 2014 | Torres, R., & Nichols, J. (2014). Health literacy knowledge and experiences of associate degree nursing students: A pedagogical study. *Teaching and Learning in Nursing*, *9*(2), 84–92. <https://doi.org/10.1016/j.teln.2013.11.003> |
| Trujillo | 2015 | Trujillo, J. M., & Figler, T. A. (2015). Teaching and learning health literacy in a doctor of pharmacy program. *American Journal of Pharmaceutical Education*, *79*(2). <https://doi.org/10.5688/ajpe79227> |
| Turner | 2009 | Turner, T., Cull, W. L., Bayldon, B., Klass, P., Sanders, L. M., Frintner, M. P., Abrams, M. A., & Dreyer, B. (2009). Pediatricians and health literacy: descriptive results from a national survey. *Pediatrics*, 124 Suppl 3, S299–S305. <https://doi.org/10.1542/peds.2009-1162F> |
| Van Der Giessen, | 2021 | Van Der Giessen, J. A. M., Van Dulmen, S., Velthuizen, M. E., Van Den Muijsenbergh, M. E. T. C., Van Engelen, K., Collée, M., Van Dalen, T., Aalfs, C. M., Hooning, M. J., Spreeuwenberg, P. M. M., Fransen, M. P., & Ausems, M. G. E. M. (2021). Effect of a health literacy training program for surgical oncologists and specialized nurses on disparities in referral to breast cancer genetic testing. *The Breast*, *58*, 80–87. <https://doi.org/10.1016/j.breast.2021.04.008> |
| Vargas | 2014 | Vargas, C. R., Chuang, D. J., & Lee, B. T. (2014). Assessment of patient health literacy: A national survey of plastic surgeons. *Plastic and Reconstructive Surgery*, *134*(6). <https://doi.org/10.1097/PRS.0000000000000737> |
| Wahab | 2018 | Wahab, A., Ali, A., Nazir, S., Ochoa, L., Khan, H., Khan, M., Chaudhary, S., & Smith, S. J. (2018). A QI initiative for bridging the health literacy gap by Educating internal medicine residents at a community hospital. *Journal of Community Hospital Internal Medicine Perspectives*, *8*(5). |
| Walker | 2019 | Walker D., Howe C., Dunkerley M., Deupree J., & Cormier C. (2019). The HLKES-2: Revision and Evaluation of the Health Literacy Knowledge and Experiences Survey. *The Journal of Nursing Education*, *58*(2), 86–92. Embase. <https://doi.org/10.3928/01484834-20190122-05> |
| Wilcoxen | 2013 | Wilcoxen, K., & King, S. R. (2013). An educational strategy to enhance pharmacy students’ attitudes toward addressing health literacy of patients. *Currents in Pharmacy Teaching and Learning*, *5*(2), 85–92. <https://doi.org/10.1016/j.cptl.2012.11.001> |
| Wittenberg | 2018 | Wittenberg E., Reb A., & Kanter E. (2018). Communicating with Patients and Families Around Difficult Topics in Cancer Care Using the COMFORT Communication Curriculum. *Seminars in Oncology Nursing*, *34*(3). <https://doi.org/10.1016/j.soncn.2018.06.007> |
| Wood | 2023 | Wood, H., Brand, G., Clifford, R., Kado, S., Lee, K., & Seubert, L. (2023). Student Health and Social Care Professionals’ Health Literacy Knowledge: An Exploratory Study. *Pharmacy (Basel, Switzerland)*, *11*(2). <https://doi.org/10.3390/pharmacy11020040> |
| Wright | 2023 | Wright, K. M. (2018). Improving Health Literacy Assessments in Pediatrics. [Walden University] |
| Wu | 2020 | Wu, S., Jackson, N., Larson, S., & Ward, K. T. (2020). Teaching geriatrics and transitions of care to internal medicine resident physicians. *Geriatrics (Switzerland)*, *5*(4), 1–14. https://doi.org/10.3390/geriatrics5040072 |
| Yang | 2022 | Yang, Y. (2022). Effects of health literacy competencies on patient-centered care among nurses. *BMC Health Services Research*, *22*(1), 1172. <https://doi.org/10.1186/s12913-022-08550-w> |
| Yogurtcu | 2022 | Yogurtcu, H., & Ozturk Haney, M. (2022). The relationship between e-health literacy and health-promoting behaviors of Turkish hospital nurses. *Global Health Promotion*, *101497462*, <https://doi.org/10.1177/17579759221093389> |

**Supplementary Table 2.** Measures used in multiple studies

| **Name of measure and/or first author** | **Number of studies** | **References** |
| --- | --- | --- |
| **Health literacy knowledge and experience survey (HLKES)**  **(Cormier 2009)** | | |
| Original | 8 | (Borrero, 2018; Cafiero, 2013; Hartman, 2014; Kennard, 2016; Kerr, 2016; Knight, 2011; Torres & Nichols, 2014) |
| Modified | 4 | (Cooper et al., 2015; Koduah et al., 2021; Maduramente et al., 2019) |
| Culturally adapted and translated | 3 | (Chang et al., 2020; Nesari et al., 2019; Parandeh et al., 2020) |
| **Health literacy knowledge and experience survey 2 (HLKES-2)**  **(Walker 2019)** | | |
| Culturally adapted and translated | 3 | (Al-Fayyadh et al., 2022; Angelopoulou et al., 2023; Haney & Yogurtcu, 2023) |
| **Nursing professional health literacy questionnaire  (Macabasco-O’Connel 2011)** | | |
| Modified | 2 | (Kim et al., 2024; Murphy & Jester, 2023) |
| Culturally adapted and translated | 1 | (Nantsupawat et al., 2020) |
| **Centre of Ethnicity and Health** | | |
| Original | 2 | (Bird et al., 2022; Martin et al., 2023) |
| **Health literacy strategies behavioural intentions questionnaire**  **(Cafiero 2013)** | | |
| Original | 1 | (Anselmann et al., 2024) |
| **Instrument of Health literacy competencies**  **(Chang 2017)** | | |
| Modified | 1 | (Hsieh et al., 2022) |
| **Cailor 2015** |  |  |
| Modified |  | (Kershner et al., 2024) |
| **Chang 2021** |  |  |
| Modified | 1 | (Chang et al., 2023) |
| **Kaper 2019** |  |  |
| Original | 1 | (Kaper, Winter, et al., 2019) |
| Modified | 1 | (Boonstra et al., 2024) |
| **Mackert 2011** |  |  |
| Original | 3 | (Coleman & Fromer, 2015; Coleman et al., 2016; Nepps et al., 2023) |
| Modified | 4 | (Coleman et al., 2017; Griffeth et al., 2022; Kaper et al., 2018; Papa et al., 2023) |
| Culturally adapted and translated | 1 | (Yogurtcu & Ozturk Haney, 2022) |
| **Rajah 2017** |  |  |
| Modified | 1 | (Mohamed-Yassin et al., 2023) |
| **Schwartzberg 2007** |  |  |
| Modified | 1 | (Payne, 2009) |
| **Sicat 2005** |  |  |
| Modified | 1 | (Potter, 2017) |

|  | **Supplementary Table 3.** Domains of health literacy competencies assessed by each unique measure | | | | | | | | |
| --- | --- | --- | --- | --- | --- | --- | --- | --- | --- |
|  | |  | Domain of health literacy | | | | | | |
| **First author and year** | | **Name of measure** | **Knowledge (performance-based)** | **Knowledge (self-report)** | **Self-efficacy and self-reported skills** | **Skills (performance-based)** | **Frequency of using health literacy techniques** | **Perceived effectiveness, perceptions and attitudes** | **Behavioural intentions** |
| Cafiero 2013 | | Health literacy strategies behavioural intentions survey (HLSBIQ) |  |  | X |  |  | X | X |
| Chang 2017 | | Instrument of health literacy competencies | X |  | X |  |  | X |  |
| Cormier 2009 | | Health literacy knowledge and experience survey (HLKES) | X |  |  |  | X |  |  |
| DeBello 2016 | | Health literacy knowledge application and confidence scale | X |  | X |  | X |  |  |
| Evans 2014 | | Health literacy beliefs and attitudes survey |  | X | X |  |  |  |  |
| Harrington 2016 | | Health literacy perception and experience survey |  |  |  |  |  | X |  |
| Jukkala 2009 | | Limited health literacy impact measure | X |  |  |  |  |  |  |
| Macabasco-O’Connel 2011 | | Nursing professional health literacy instrument | X |  |  |  | X | X |  |
| Munangatire 2022 | | Understanding of health literacy questionnaire | X |  |  |  |  |  |  |
| Naperola-Johnson 2022 | | Health literacy knowledge check | X |  |  |  | X | X |  |
| Newton 2018 | | Nurse knowledge survey |  |  |  |  |  |  |  |
| Ogrodnick 2021 | | Health literacy beliefs and knowledge survey | X |  |  |  |  | X |  |
| O’Neal 2013 | | AHRQ health literacy assessment tool |  | X |  |  |  |  |  |
| Palesy 2020 | | Homecare worker health literacy scale |  |  | X |  |  |  |  |
| Walker 2009 | | Health literacy knowledge and experience survey 2 (HLKES-2) | X |  |  |  | X |  |  |
| Wu 2020 | | Carolina geriatrics education centre health literacy survey |  | X | X | X |  | X |  |
| Ali 2014 | |  |  | X | X |  |  |  |  |
| Anwar 2019 | |  | X |  | X |  | X | X |  |
| Baldocchi 2013 | |  |  |  | X |  | X |  |  |
| Billek-Sawhney 2012 | |  | X |  |  |  |  |  |  |
| Bilotta 2012 | |  | X |  |  |  | X |  |  |
| Bird 2022 | |  |  | X | X |  |  |  |  |
| Cailor 2015 | |  | X | X | X |  |  | X |  |
| Chang 2021 | |  |  | X | X |  |  | X |  |
| Costich 2022 | |  |  |  |  | X | X |  |  |
| D’Abbondanza 2023 | |  |  | X | X |  | X | X |  |
| Devraj 2010 | |  | X |  | X |  |  |  |  |
| Devraj 2011 | |  |  |  |  |  |  | X |  |
| Devraj 2012 | |  | X |  |  |  |  |  |  |
| Faruqi 2015 | |  |  |  |  |  | X |  |  |
| Feinberg 2021 | |  | X |  |  |  |  | X |  |
| Fetene 2024 | |  | X |  |  |  |  |  |  |
| Finlay 2019 | |  | X |  |  |  |  |  |  |
| Galati 2018 | |  |  | X | X |  | X |  |  |
| Gibson 2022 | |  |  | X | X |  | X |  |  |
| Flick 2023 | |  |  |  |  |  | X |  |  |
| Goto 2014 | |  |  | X | X |  |  |  |  |
| Goto 2015 | |  |  | X |  |  |  |  |  |
| Grabeel 2018 | |  | X |  | X |  |  | X |  |
| Green 2014 | |  | X |  | X | X |  | X |  |
| Groene 2017 | |  | X |  | X |  |  | X |  |
| Guner 2019 | |  |  |  |  |  | X |  |  |
| Gupta 2020 | |  |  |  | X |  | X |  |  |
| Ha 2014 | |  | X |  |  |  |  |  |  |
| Kaper 2019 | |  |  | X | X |  | X | X |  |
| Kelly 2020 | |  |  | X | X |  | X | X | X |
| Koenig 2019 | |  | X |  | X |  |  | X | X |
| Logan 2007 | |  |  |  |  |  |  | X |  |
| Mackert 2011 | |  |  | X | X |  | X |  |  |
| McCleary-Jones 2012 | |  | X |  |  |  |  |  |  |
| McCune 2010 | |  | X |  | X |  | X |  |  |
| Mibei 2018 | |  | X |  |  |  | X |  |  |
| Mihalopoulos 2013 | |  | X |  |  |  | X |  |  |
| Milford 2016 | |  |  | X | X |  |  | X |  |
| Mnatzaganian 2017 | |  | X |  |  |  |  | X |  |
| Muscat 2021 | |  |  |  | X |  |  |  | X |
| Ogrodnick 2020 | |  | X |  |  |  | X | X |  |
| Pagels 2015 | |  | X |  | X |  |  | X |  |
| Rajah 2017 | |  | X |  |  |  |  | X |  |
| Roberts 2012 | |  | X |  | X |  |  |  |  |
| Ronan 2023 | |  |  |  |  |  |  | X |  |
| Ruggeri 2021 | |  | X |  |  |  |  |  |  |
| Sand-Jecklin 2010 | |  | X |  |  |  |  |  |  |
| Schlichting 2007 | |  |  |  |  |  | X |  |  |
| Schwartzberg 2007 | |  |  |  |  |  | X | X |  |
| Sharifirad 2015 | |  |  |  |  |  | X | X | X |
| Shing 2023 | |  | X | X | X |  | X | X |  |
| Sicat 2005 | |  | X |  | X |  |  |  |  |
| Stone 2021 | |  |  |  | X |  |  |  |  |
| Suarez-Balcazar 2024 | |  |  | X | X |  |  |  |  |
| Sullivan 2011 | |  |  | X | X |  |  |  |  |
| Szwajcer 2014 | |  | X |  | X |  | X |  | X |
| Trujillo 2015 | |  | X |  | X |  |  | X |  |
| Turner 2009 | |  |  |  | X |  | X | X |  |
| Van Der Giessen 2021 | |  | X | X | X |  |  | X |  |
| Vargas 2014 | |  | X |  |  |  | X | X |  |
| Wahab 2018 | |  | X |  |  |  |  |  |  |
| Wilcoxen 2013 | |  |  |  | X |  |  | X | X |
| Wittenberg 2018 | |  |  |  | X |  | X |  |  |
| Wood 2023 | |  | X |  |  |  |  |  |  |
| Wright 2018 | |  | X |  |  |  |  |  |  |
| Yang 2022 | |  | X |  | X |  |  |  |  |

|  | **Type of validity testing** | | | | |
| --- | --- | --- | --- | --- | --- |
| **Measure (first author)** | **Face validity** | **Content validity** | **Construct validity** | **Criterion validity** | **Reliability testing** |
| Anselmann 2024 |  |  |  |  | X |
| Anwar 2019 | X | X |  |  | X |
| Bilotta 2019 |  | X |  |  | X |
| Cailor 2015 |  |  |  |  | X |
| Chang 2017 |  |  | X |  | X |
| Chang 2021 |  | X | X |  | X |
| Congying 2024 |  |  | X |  | X |
| Costitch 2022 |  |  |  |  | X |
| Cormier 2009 |  | X |  |  | X |
| DeBello 2016 |  |  | X |  | X |
| Devraj 2011 |  | X |  |  | X |
| Devraj 2012 |  |  |  |  | X |
| Feinberg 2021 | X |  |  |  | X |
| Galati 2018 | X | X |  |  | X |
| Gibson 2022 |  | X |  |  |  |
| Guner 2019 |  | X |  |  | X |
| Hartmann 2014 | X | X |  |  | X |
| Kaper 2019 |  |  |  |  | X |
| Kelly 2020 |  | X |  |  | X |
| Koenig 2019 |  |  |  |  | X |
| Logan 2007 |  |  |  |  | X |
| Macabasco-O’Connell 2011 |  | X |  |  |  |
| McCleary-Jones 2012 | X |  |  |  | X |
| McCune 2013 |  |  |  |  | X |
| Mibei 2019 |  |  |  |  | X |
| Mihalopolos 2013 |  | X |  |  |  |
| Munangatire 2022 |  | X | X |  | X |
| Ogrodnick 2020 |  |  |  |  | X |
| O’Neal 2013 |  | X |  |  |  |
| Potter 2017 |  |  |  |  | X |
| Rajah 2017 |  | X |  |  | X |
| Schlichting 2021 | X |  |  |  |  |
| Sharifirad 2015 |  | X |  |  | X |
| Walker 2019 | X | X |  |  | X |
| Wilcoxen 2013 | X |  |  |  | X |
| Wu 2020 |  |  |  |  | X |
| Yang 2022 |  | X |  |  | X |

**Supplementary Table 4.** Reported validity and reliability testing of unique measures (n=37)

**Supplementary Table 5.** Validation and reliability testing methods of unique measures (n=37)

| **Type of validation or reliability testing** | **Methods** | **n (%)** | **Reference** |
| --- | --- | --- | --- |
| **Validation** |  |  |  |
| Content validity | Expert review | 13 (35) | (Anwar et al., 2019; Bilotta, 2012; Gibson et al., 2022; Guner & Ekmekci, 2019; Gupta et al., 2020; Hartman, 2014; Kelly et al., 2020; Macabasco-O’Connell et al., 2011; Munangatire et al., 2022; O’Neal et al., 2013; Rajah et al., 2017; Sharifirad et al., 2015; Walker et al., 2019) |
|  | Pilot testing | 5 (14) | Anwar et al., 2019; Devraj et al., 2011; Galati et al., 2018; Mihalopoulos et al., 2013; Munangatire et al., 2022) |
|  | Content validity index (CVI) | 3 (8) | (Chang et al., 2021; Cormier 2009; Yang, 2022) |
|  | Content validity ratio (CVR) | 1 (3) | (Sharifirad 2015) |
| Face validity | Expert review | 8 (22) | (Anwar et al., 2019; Bilotta, 2012; DeBello, 2016; Gibson et al., 2022; Rajah et al., 2017; Ronan et al., 2023; Sharifirad et al., 2015; Wittenberg et al., 2018) |
|  | Pilot testing | 4 (11) | (Anwar et al., 2019; Galati et al., 2018; Hartman, 2014; Wilcoxen & King, 2013) |
|  | Cognitive interviews | 1 (3) | (Walker et al., 2019) |
|  | Test blueprint | 1 (3) | (McCleary-Jones, 2012) |
|  | Not reported | 2 (5) | (Feinberg et al., 2021; Schlichting et al., 2007) |
| Construct validity | Factor analysis (Exploratory or Confirmatory Factor analysis) | 2 (5) | (Chang et al., 2021; Munangatire et al., 2022) |
|  | Principal component analysis | 2 (5) | (DeBello, 2016; Devraj 2011) |
|  | Item response theory | 1 (3) | (Chang 2017) |
|  | Bartletts test | 1 (3) | (Congying et al., 2024) |
| **Reliability** |  |  |  |
| Internal consistency | Cronbach’s alpha | 28 (76) | (Anselmann et al., 2024; Anwar et al., 2021; Bilotta, 2012; Cailor & Chen, 2015; Chang et al., 2017; Chang et al., 2021; Congying et al., 2024; Cormier & Kotrlik, 2009; DeBello, 2016; Devraj & Gupchup, 2011; Galati et al., 2018; Guner & Ekmekci, 2019; Hartman, 2014; Kaper et al., 2019; Kelly et al., 2020; Koenig & Provident, 2019; McCleary-Jones, 2012; McCune, 2010; Mibei & Daniels, 2018; Munangatire et al., 2022; Ogrodnick et al., 2020; Potter, 2017; Rajah et al., 2017; Sharifirad et al., 2015; Walker et al., 2019; Wilcoxen & King, 2013; Wu et al., 2020; Yang, 2022) |
|  | Kuder Richardson coefficient | 1 (3) | (Devraj & Gupchup, 2012) |
| Inter-rater reliability | Cohen’s kappa | 2 (5) | (Costich et al., 2022; Logan, 2007) |
| Test-retest reliability | Intraclass correlation coefficient | 1 (3) | (Feinberg et al., 2006) |
